# Flame retardants of the future: biobased, organophosphorus, reactive or oligomeric

**DOI:** 10.3389/fchem.2024.1500782

**Published:** 2024-11-01

**Authors:** Bob A. Howell

**Affiliations:** Science of Advanced Materials, Center for Applications in Polymer Science, Department of Chemistry and Biochemistry, Central Michigan University, Mt. Pleasant, MI, United States

**Keywords:** structural control of flame retardant acrion, non-toxic flame retardants, non-migrating flame retardants, oligomeric flame retardants, highly branched flame retardants, sustainable flame retardants

## Abstract

Polymeric materials have been a great boon to the development and wellbeing of mankind. However, in the main, these materials are flammable and must be flame retarded for most applications. Many substances have been utilized to impart a measure of flame retardancy. The most widely used and most effective have been organic: organohalogen and organophosphorus compounds. Organohalogen compounds have been popular, low-cost, very effective flame retardants for polymeric materials. However, with the recognition that these compounds readily migrate from a polymer matrix into which they have been incorporated, persist in the environment and pose serious risks to human health, the use of organophosphorus compounds has become prominent. In particular, organophosphorus compounds of appropriate structure derived from readily-available, renewable, low-cost, non-toxic biobased precursors are attractive. Avoidance of the issues of environmental persistence and toxicity associated with organohalogen compounds is possible with these materials. Migration from a polymer matrix may be removed as a deficiency through the use of reactive compounds, i.e., compounds that may be incorporated directly into the polymer structure either by copolymerization or grafting, or oligomeric compounds. Oligomeric materials of branched structure display characteristics of broad compatibility, high effectiveness and lack of migration.

## Introduction

Polymeric materials have positively impacted human societies from the earliest times. These early materials were poly(saccharide)s or poly(amide)s of natural plant or animal origin. Even these early materials needed to be flame retarded for safe use. An early example is the attempt by Gay-Lussae to flame retard theater curtains using a mixture of alum, ammonium salts and vinegar (Gay-Lussac, 1821). With the advent of the modern polymer industry in the middle of the last century, the use of flame retardants became critical. In the late 1920s, Herman Staudinger correctly perceived that macromolecular materials were polymers and fundamentally altered the understanding of these materials (Furukawa, 1998). Within a few short years, Wallace Carothers working at DuPont had established a strong experimental base for these observations (Furukawa, 1998; Hermes, 1996; Hounshell and Smith, 1988). Nylon stockings were unveiled at the 1939 world’s fair in New York and were a sensation—nylon 6,6 was the new silk. More importantly, this new material was crucial to the manufacture of parachutes which made possible the allied landing at Normandy in 1944. During that period, poly(ethylene) was discovered in England and played a crucial role as coating for radar cable (Perrin, 1953). After the war, the polymer industry developed rapidly and the use of flame retardants became necessary and widespread (Factor, 1974; Pritchard, 1998; Lyons, 1970).

## Development of flame retardants

Early flame retardants were largely inorganic in nature. Some are still in use today in applications for which polymer integrity is not important. Generally, these are metal oxides or hydroxides (aluminum, magnesium, boron, etc.) often as hydrates. These materials were chosen empirically and were not highly effective. Neither the process for polymer combustion or flame retardant action were understood. Almost any polymeric material when heated to a sufficiently high temperature will begin to thermally degrade to generate small volatile fragments which escape to the gas phase. In the gas phase these fragments mix with oxygen and, if the temperature is appropriate—at or above the ignition temperature—combustion may occur. Combustion is an exothermic radical process which liberates heat and light (flame) and, if combustion is incomplete, particulates (soot, smoke). Heat generated may feedback to the polymer surface and enhance the rate of degradation and consequently the supply of fuel fragments to the combustion zone (Howell et al., 2022). Consequently, there are two opportunities to inhibit flammability. The first is in the solid phase, i.e., to retard polymer pyrolysis and reduce fuel evolution to the gas phase. A preponderance of flame retardants function in this way. They are either sufficiently acidic or decompose in the degrading polymer matrix to generate acidic substances which promote cationic crosslinking and the formation of a char layer at the surface of the degrading polymer. This char layer acts as an insulation barrier to inhibit heat feedback from the combustion zone. This reduces the rate of fuel formation to support combustion. The second is in the gas phase. Some flame retardant additives, including many inorganic compounds, nitrogen-containing substances, and others decompose to form volatile inert species which escape to the gas phase and dilute the fuel load in the combustion zone. More effective agents decompose in the polymer matrix to form active species capable of avoiding complete reaction with matrix components and volatilization to the gas phase where they may effectively interrupt the radical combustion process.

Most inorganic flame retardants, as noted, function by decomposition to liberate inert fragments, often water, to the gas phase to dilute the fuel load in the combustion zone. Consequently, they are not very efficient and large amounts are required to introduce an acceptable level of flame retardancy. Large filler loadings often diminish polymer physical properties such that they are unsuitable for many applications. Most often these materials are used in polymers intended for paper or cable coating for which polymer integrity is relatively unimportant. The more effective flame retardants have been organic compounds, either organohalogen or organophosphorus (Factor, 1974; Pritchard, 1998; Lyons, 1970). Organohalogen flame retardants were developed first. On an elemental basis alone, halogens, either chlorine or bromine, are much less expensive than phosphorus. Further, phosphorus compounds are in great demand in several other areas from fertilizers to food additives. The first organohalogen flame retardants were chloroalkanes. These had some severe limitations. They tended to decompose at temperatures below the degradation temperature of many polymers. Thus, although they efficiently liberate active hydrogen chloride to the gas phase much is lost before fuel for combustion is being formed. Perhaps, the most prominent of the organochlorine flame retardants was Derchlorane Plus, the *bis*-Diels-Alder adduct of hexachlorocyclopentadiene with cyclooctadiene. These compounds display significant toxicity and became widespread environmental pollutants (Sverko et al., 2011). They were largely replaced by organobromine compounds. Aliphatic bromine compounds undergo thermally-induced dehydrobromination at temperatures too low to be useful flame retardants, with a few exceptions, e.g., foamed poly(styrene), for most polymeric materials. However, brominated aromatic compounds, owing to the greater thermal stability of aromatic-bromine bonds, undergo decomposition in the temperature range for which many polymers degrade. Bromoaromatics, particularly brominated aryl ethers, have been widely utilized as cost effective and efficient flame retardants. Diphenyl ether is readily available as a byproduct of phenol production (Wittcoff et al., 2012). It can be easily converted to bromo derivatives with flame retarding properties. The most widely used has been decabromodiphenyl ether (Alaee et al., 2003). The effectiveness of these compounds may be augmented by the presence of a metal oxide in the degrading polymer matrix. The metal oxide gets converted to volatile metal halides or oxyhalides which escape to the gas phase to interrupt combustion propagation reactions. These materials are not synergists, i.e., they do not impact the decomposition of the primary flame retardant. They are precursors to flame retardants, the corresponding metal halides. The impacts of the primary flame retardant and the metal halide occur in parallel, i.e., one does not affect the other. The overall result is cooperative action which leads to greater overall flame retardancy (more effective utilization of bromine). Although several metal halides, depending on volatility, may act in this way, antimony tribromide is the most effective. Consequently antimony oxide has commonly been used as an additive with organobromine flame retardants. Antimony oxide plays a similar role as an additive for poly(vinyl chloride) [PVC]. Unformulated PVC is effectively non-flammable (LOI 45). However, for processing and use PVC must be heavily plasticized. The incorporation of large amounts of phthalate plasticizer introduces two problems: toxicity and flammability. Flammability may be controlled by the incorporation of antimony oxide during the processing. As the polymer encounters high temperatures and the polymer begins to degrade and plasticizer to be volatilized, antimony chlorides are formed and enter the gas phase where they function as efficient flame retardants. Because of the toxicity (and cost) of antimony oxide other precursors to volatile metal halides have been sought (Howell et al., 2020).

While the brominated aryl ethers function as effective flame retardants, they display several negative features. They tend to migrate from a polymer matrix into which they have been incorporated and enter the environment. Further, at high temperature, as in a fire, they are converted to volatile, very toxic dioxins and furans (Zhang et al., 2016). When items containing these compounds are discarded in a landfill they are leached into waterways where they bioaccumulate and may enter the human food chain (Cristale et al., 2019). Human exposure to organobromine flame retardants leads to the development of a number of disease states, most arising from endocrine disruption (Darnerud, 2008; Hamers et al., 2006; Wu et al., 2020; Sken et al., 2024). To circumvent ease of migration from a polymer matrix, environmental contamination, and human exposure, brominated substances that may incorporated into a polymer structure by copolymerization or grafting and bromine-containing oligomers have been developed (Gelmont et al., 2020; Narayan and Moore, 2012). Because of toxicity and widespread human exposure, organohalogen flame retardants have come under increasing societal and regulatory pressure. Several have been removed from the market. It is now commonplace to use mixtures of additives to achieve satisfactory flame retardance. Synergy of action has been a goal. Unfortunately, true synergy has seldom, if ever, been observed. Generally, multiple flame retardant additives function in parallel, i.e., one does not impact the function of another. Further, the impact of the presence of multiple additives is usually not additive, i.e., the overall impact does not equal the sum of the impacts of the individual agents used separately. The cooperative actions can, however, lead to enhanced overall flame retardancy. Nitrogen compounds have often been used in conjunction with organophosphates. The nitrogen compound decomposes to generate volatile inert species which dilute the fuel load in the gas phase while the phosphate decomposes to generate acidic substances to promote crosslinking and char formation in the solid phase.

## Phosphorus flame retardants

Because of the negative health effects associated with the use of organohalogen flame retardants, organophosphorus compounds have received greater attention. The flame retarding properties of organophosphorus compounds have long been known (Granzow, 1978). With a need to find replacements for popular but toxic organohalogen flame retardants, the interest in organophosphorus compounds has dramatically increased (Velencoso et al., 2018). Many phosphorus compounds, both inorganic and organic, have been utilized in flame retarding applications. Early flame retardants were simple phosphate salts, particularly ammonium, or esters. These were generally solid-phase active. Polyphosphates have also been utilized as effective flame retardants (Mauldin et al., 2014). Polyphosphate flame retardants have been commercialized (FRX Innovations and Inc., 2024). A major development has been the advent of 9,10-dihydro-9-oxa-10-phosphaphenanthrene-10-oxide (DOPO) as a flame retardant both because of its effectiveness and what it illustrates about the structural requirements for flame retardancy (Howell, 2022). Because of its unique structure, there is a strong thermodynamic driving force (formation of a stable dibenzofuran) for thermal decomposition to extrude the PO radical as well as an accessible pathway for reaction (Schafer et al., 2014; Liang et al., 2015). The PO radical lacks sufficient reactivity to be completely consumed by elements of a degrading polymer matrix such that a portion of that formed is evolved to the gas phase where it efficiently scavenges combustion propagating radicals. This is in contrast to the behavior of most organophosphorus compounds. Most are solid-phase active. The mode of flame retardant action for organophosphorus compounds is strongly dependent on the level of oxygenation at phosphorus (Howell, 2022). Those with a high level of oxygenation at phosphorus, e.g., phosphates, undergo thermal decomposition at relatively low temperatures to form acidic species which promote cationic crosslinking in the degrading polymer matrix and formation of a char layer at the surface. This char layer acts as an insulation barrier to inhibit heat feedback from the combustion zone and retard formation of volatile fuel fragments. Those with low levels of oxygenation at phosphorus, e.g., phosphonates, phosphinates, phosphine oxides, undergo decomposition in a degrading polymer to ultimately form the PO radical. Evolution of the PO radical to the gas phase interrupts radical processes in the combustion zone. The formation of other active volatile phosphorus radical species has sometimes been suggested but this has yet to be rigorously demonstrated.

## Biobased organophosphorus flame retardants

Several developments over the past decade have propelled biobased organophosphorus flame retardants to a position of prominence as potential useful polymer additives. An obvious impetus for interest in these materials has been the recognition of the environmental persistence and toxicity of organohalogen flame retardants. Further, biobased organophosphorus compounds may be derived from abundantly-available, renewable, non-toxic precursors. Often these precursors are available as under-utilized byproducts of major industrial processes or from the natural biopolymers, starch or cellulose (Howell et al., 2018; Howell et al., 2021; Han et al., 2022). Prominent among these might be tartaric acid (from wine-making), cardanol (from cashew shell liquid), diphenolic acid, tannic acid, gallic acid, grain phenolics and isosorbide and the furanics (from starch or cellulose). Isosorbide, a carbohydrate derivative, is particularly useful for the production of effective organophosphorus flame retardants (Howell and Daniel, 2020). In particular, the DOPO adduct of isosorbide *bis*-acrylate which undergoes thermal decomposition above 300°C offers potential as a flame retardant for polymers that process at high temperatures (Daniel and Howell, 2018). Furanics, too, provide a biobase for the generation of flame retardants (Howell and Han, 2020). In the main, phosphorus compounds generated from biobased precursors display low toxicity (Howell, 2024). In particular, DOPO derivatives exhibit little toxicity (Hirsch et al., 2017; Liu et al., 2018). Biobased organophosphorus flame retardants not only have the potential to replace toxic organohalogen flame retardants but to provide superior effectiveness in the absence of environmental accumulation and excessive human toxicity.

## Development of flame retardants for the future

New flame retardants must meet requirements for effectiveness, environmental compatibility and sustainable production. These materials must be available by simple, low cost processes, be widely compatible with polymeric materials and be readily destroyed during polymer recycling. Above all, they must be non-migrating and non-toxic. Organophosphorus compounds derived from, appropriately selected, biobased precursors have the potential to meet all these criteria. Not only is the selection of biobased substrate important but so too is the structure of the phosphorus moiety incorporated. The most effective organophosphorus flame retardants are those containing a low level of oxygenation at phosphorus and having a structure conducive to extrusion of a PO radical turning thermal decomposition (Schafer et al., 2014; Liang et al., 2015; Howell, 2022). The PO radical is not sufficiently reactive (most radicals generated during thermal decomposition of organophosphorus compounds are rapidly consumed by reaction with components of the degrading polymer matrix) to be completely consumed by reaction in the polymer matrix. Some of the PO radicals formed escape to the gas phase where they efficiently scavenge flame propagating radicals. This is in contrast to most phosphorus agents which decompose to form phosphorus acids that prompt cationic crosslinking and char formation in the degrading polymer matrix. The structure of the phosphorus moiety is crucial for maximal flame retardant activity—a pathway for the formation of the PO radical must be available. It is not sufficient to just get phosphorus species into the gas phase. To display flame retardant activity they must be capable of efficient reaction with combustion propagating radicals. The selection of an appropriate structure is a first requirement for the synthesis of an effective organophosphorus flame retardant.

The mode of action and toxicity of an organophosphorus flame retardant may be determined by careful design of structural features. Migration potential may be controlled in two ways. The use of organophosphorus additive that may be covalently incorporated into the polymer structure, i.e., “reactive” flame retardants, permits effective flame retardance with no possibility of migration (Stovall and Long, 2024). For example, incorporation of small amounts of a phosphorus-containing isosorbide acrylate into a styrene polymer imparts good flame retardancy (Howell and Daniel, 2019). A second approach is to use the polymeric flame retardants. The most effective contain polar groups and a high degree of branching to facilitate compatibility with a wide range of polymeric materials. Phosphorus-containing hyperbranched poly(ester)s offer great potential in this area. Hyperbranched poly(ester)s may be prepared from naturally-occurring polyols and diacids. Glycerol is a trifunctional alcohol abundantly-available from soap-making and biodiesel manufacture (Tan et al., 2013). As a consequence of trifunctionality, condensation of glycerol with a natural diacid usually results in the formation of a highly crosslinked, gelled structure. Traditionally, empirical methods, most often limited monomer conversion, has been employed in an attempt to avoid gelation (Sumbe and Bruckmann, 2004; Wyatt, 2012). However, these materials contain two kinds of endgroups, both hydroxyl and carboxyl, and will gel on storage. Now, newly developed technology, the Martin-Smith model for determining monomer ratios, permits polymerization to high conversion without gellation to generate hyperbranched oligomers of any desired molecular weight, well-defined structure and a single kind of endgroup (Zhang et al., 2017; Zhang et al., 2014). These materials contain ester functionality, a high degree of branching and a large number of endgroups. All these features enhance compatibility with a polymer matrix. However, it is the presence of a single kind of endgroup that makes them attractive for the generation of effective flame retardants. For example, an oligomeric hydroxyl terminal ester may be readily converted, via a simple esterification, to a material with multiple phosphorus-containing entities. Great versatility is possible. For example, the presence of phosphate endgroups will afford a solid-phase active flame retardant while that containing phosphonate endgroups will function as a gas-phase flame retardant. A mixture of the two will provide both kinds of flame retardant activity. Perhaps, more impressive would be the generation of a hyperbranched oligomer from a phosphorus-containing core. For example, preparation of an oligomer from trimethyl phosphate and ethylene glycol followed by capping as a DOPO ester would provide a material with both solid-phase and gas-phase activity.

## Conclusion

The demise of organohalogen flame retardants has created a period of uncertainty and a focus on short-term solutions within the flame retardant community. Mixtures of additives to achieve satisfactory flame retardancy are constantly being sought. This and other temporary measures do not address the longer term need for effective, accessible, widely-applicable, non-toxic flame retardants. Mode of action and toxicity may be controlled through proper choice of structure for organophosphorus compounds. Migration from a polymer matrix may be prevented through the use of compounds that become covalently incorporated into the polymer structure or oligomeric additives. Oligomeric additives offer the greater flexibility and ease of use. Those derived from glycerol and natural diacids display particularly attractive features. It seems clear that the next-generation of broadly useful flame retardants will be biobased organophosphorus and either reactive or oligomeric.

## Data Availability

Requests to access the datasets should be directed to Bob Howell, bob.a.howell@cmich.edu.
